# Factors Associated with Prescription-to-Vaccination Conversion and Timeliness: A Real-World Analysis

**DOI:** 10.3390/vaccines14080720

**Published:** 2026-08-20

**Authors:** Mengru Xiao, Jun Li, Shejun He, Meiqiong Xiang, Xiaohong Dong, Xiaoyang Wang, Xiaoxiao Zhang, Yonghao Guo, Yanyang Zhang

**Affiliations:** 1 College of Public Health, Zhengzhou University, Zhengzhou 450001, China; xmr20011206@126.com; 2 Immunization Prevention and Planning Institute, Henan Provincial Center for Disease Control and Prevention, Zhengzhou 450016, China; henan_epi2@163.com (J.L.); wxy6424@163.com (X.W.); xiao5541@126.com (X.Z.); cdcgyh@163.com (Y.G.); 3 Henan Academy of Preventive Medicine, Zhengzhou 450016, China; 4 Lingbao Center for Disease Control and Prevention, Lingbao 472500, China; hsj158@163.com (S.H.); hnlbxmq@126.com (M.X.); dxh6535@126.com (X.D.)

**Keywords:** vaccine prescription, conversion rate, conversion timeliness, associated factors, real-world study

## Abstract

Background: This study evaluated vaccine prescription conversion and identified associated factors in Henan Province, China, during 2025. Methods: The study was conducted in Lingbao City, Henan Province. All vaccine prescriptions issued in 2025 were collected, and vaccination status was linked through the Henan Provincial Immunization Information Management System. Subgroup analyses were stratified by vaccine type (NIP vs. non-NIP). Univariate analyses used χ^2^, Mann–Whitney U, and Kruskal–Wallis H tests. Multivariable analyses employed Firth-corrected logistic regression for conversion behavior, and mixed-effects models with random intercepts for patient ID for conversion timeliness, analyzed using a two-part model. All analyses were performed using R software (version 4.5.3). Results: Among 50,887 prescriptions, the overall conversion rate was 13.8%. NIP vaccines had a higher conversion rate than non-NIP vaccines (76.8% vs. 12.5%, *p* < 0.001) and a higher same-day conversion rate (13.9% vs. 7.4%; χ^2^ = 59.585, *p* < 0.001), but longer delays among non-same-day conversions (23 vs. 4 days). Manual prescriptions were associated with significantly higher conversion in both NIP (OR = 3.913) and non-NIP (OR = 37.720) subgroups. Vaccination clinics were associated with a nearly 20-fold higher odds (OR = 19.386). Interaction analyses showed manual prescriptions were more strongly associated with conversion and shorter delays in older adults, and had stronger associations with conversion for non-NIP vaccines (χ^2^ = 22.275, *p* < 0.001). Vaccination clinics were most strongly associated with conversion in children (Δ*P* = 0.130); however, the improvement in same-day conversion among older adults was relatively smaller, suggesting additional barriers beyond clinic availability may exist in this population. Conclusions: Manual prescriptions and vaccination clinics were associated with higher vaccine prescription conversion, with particularly pronounced effects on non-NIP vaccines. Older adults exhibited the lowest conversion rates. In parallel with promoting electronic prescriptions, co-issuing paper prescriptions is recommended, particularly for non-NIP vaccines and older adults.

## 1. Introduction

Recognized by the World Health Organization as one of the most successful public health interventions, immunization has saved an estimated 154 million lives over the past 50 years [1]. In China, the “Healthy China 2030” Planning Outline explicitly endorses a “prevention first” strategy, calling for a universal public health service system covering the entire population across the life course [2]. This policy direction is reinforced by the Guiding Opinions of the General Office of the State Council on Promoting High-Quality Development of Disease Prevention and Control, which identify vaccination as a key component of public health intervention capacity and emphasize the optimization of service models and expansion of coverage [3]. Within the National Immunization Program (NIP), childhood vaccination coverage in China has consistently remained at high levels, resulting in a marked decline in the incidence of vaccine-preventable diseases [4]. However, substantial gaps persist in vaccination coverage among the general adult population, particularly among older adults and individuals with chronic conditions [5]. Non-NIP vaccines are voluntary and self-paid [6], requiring longer decision-making by recipients and generally yielding lower coverage rates. Their uptake is constrained by multiple factors, including limited public awareness, vaccine hesitancy, financial burden, and poor accessibility to vaccination services [7].

Against this backdrop, the “vaccine prescription” model—which integrates vaccination recommendations into routine clinical practice—has gradually gained policy attention. The core approach involves clinicians proactively offering vaccination recommendations or issuing prescriptions to eligible patients during clinical encounters, thereby shifting vaccination decisions from “passive waiting” to “active guidance” and advancing the integration of medical and preventive services [8,9]. Since 2023, Qingdao City has served as a provincial pilot site, pioneering an adult vaccine prescription model in which clinicians assess vaccine-preventable disease risks based on patients’ age, underlying conditions, and immunization history to issue personalized vaccine prescriptions. By May 2026, 235 healthcare institutions in Qingdao had issued approximately 68,000 vaccine prescriptions, with 24,000 doses administered, yielding a prescription conversion rate of 35%, according to the Qingdao Municipal Health Commission [10]. Similarly, Weifang City has empowered community physicians to prescribe vaccines, significantly improving public vaccination willingness [11]. These pilot experiences suggest that the vaccine prescription model is feasible and promising. However, the conversion process from prescription issuance to actual vaccination is influenced by multiple factors—including prescription format, vaccination clinic availability, vaccine type, and recipient characteristics—and the underlying mechanisms remain insufficiently understood due to a lack of systematic empirical research.

Existing studies have largely focused on single vaccines (e.g., influenza, HPV) or specific populations (e.g., older adults, children) [12,13,14], providing limited evidence on real-world vaccine prescription conversion across diverse populations and multiple vaccine types. Moreover, NIP and non-NIP vaccines differ fundamentally in terms of target populations, payment mechanisms, and delivery models; however, direct comparative evidence on whether these differences translate into distinct conversion patterns is lacking. Therefore, leveraging real-world prescription and conversion data that simultaneously capture both NIP and non-NIP vaccines, and systematically evaluating prescription-to-vaccination conversion and timeliness and its correlates across multiple dimensions—whether vaccinated, whether vaccinated in a timely manner, and length of delay—is of substantial practical importance [15].

Lingbao City, a county-level city in Henan Province, has a well-established vaccination service network and a robust hospital information system that supports real-world data collection for vaccine prescription conversion research. This study used vaccine prescription data from Lingbao City for the full year of 2025 to analyze post-prescription conversion rates and timeliness, identify factors associated with prescription-to-vaccination conversion and timeliness, and compare differences between NIP and non-NIP vaccines. The findings are intended to inform the optimization of vaccine prescription service workflows and to improve overall prescription-to-vaccination conversion and timeliness.

## 2. Materials and Methods

### 2.1. Data Sources, Inclusion and Exclusion Criteria

This retrospective cohort study analyzed real-world vaccine prescription conversion and its associated factors. Prescription data were sourced from HIS, the Basic Public Health System, and manual records in Lingbao City, with vaccination records from the Henan Provincial Immunization Information Management System. This system provides province-wide coverage of immunization records from all vaccination clinics across Henan. Record linkage was performed using the resident identification number as the primary matching key, supplemented by name, sex, and date of birth, with an overall linkage success rate exceeding 98%. During 2025, 50,887 prescriptions were issued to 37,031 patients across 541 facilities (mean: 1.37 per patient; 75.0% had only one). The unit of analysis was the vaccine-specific prescription record. If a single prescription covered multiple vaccines, it was split into multiple vaccine-specific records. The index date was defined as the date of prescription issuance. All prescriptions issued between 1 January 2025 and 31 December 2025 were included. Follow-up began on the index date and continued until the data cutoff date of 26 April 2026. Individuals who had not been vaccinated by the end of follow-up period were censored at 26 April 2026.

**Figure 1:**
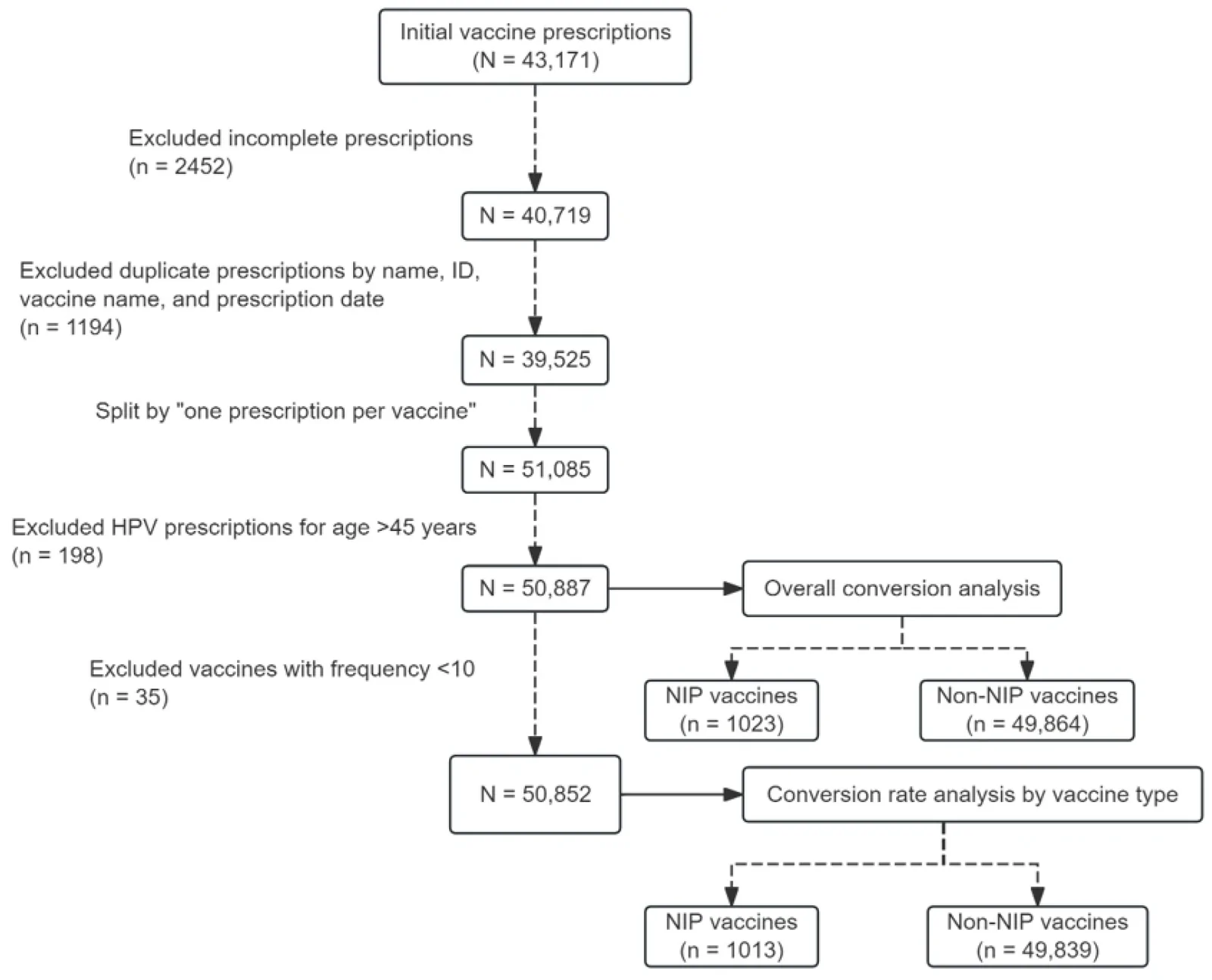
Study population selection flowchart. A total of 43,171 initial vaccine prescriptions were identified. After excluding incomplete records (*n* = 2452), duplicates (*n* = 1194), HPV prescriptions for individuals aged >45 years (*n* = 198), and vaccines with a frequency of <10 (*n* = 35), 50,852 prescriptions remained for analysis, comprising 1023 NIP vaccines and 49,864 non-NIP vaccines. Among these, 1013 NIP vaccines and 49,839 non-NIP vaccines were included in the vaccine-specific conversion rate analysis. If a single prescription covered multiple vaccines, it was split into multiple vaccine-specific records, resulting in 51,085 observations from the initial 43,171 prescriptions. NIP: National Immunization Program.

All vaccine prescriptions issued within the study period were included. Prescriptions with negative age values or missing key variables were excluded. The study population selection process is presented in Figure 1.

### 2.2. Variable Definitions

The outcome variables were defined as follows: conversion (whether a vaccine prescription was converted into actual vaccination), same-day conversion (whether the vaccination occurred on the same day the prescription was issued), and time to conversion for non-same-day conversions (the number of days from prescription issuance to vaccination).

The predictor variables were defined accordingly. Vaccine type was classified according to the National Immunization Program (NIP) vaccine catalog. Vaccines administered under the NIP schedule—including hepatitis B, BCG, polio, DTaP (diphtheria–tetanus–acellular pertussis), DT (diphtheria–tetanus), meningococcal polysaccharide vaccines (Groups A and A+C), MMR (measles–mumps–rubella), Japanese encephalitis, and hepatitis A vaccines—were classified as NIP vaccines; all others were classified as non-NIP vaccines. Prescription format was categorized as electronic prescriptions or manual prescriptions. Vaccination clinic availability was determined by the Lingbao Center for Disease Control and Prevention’s accreditation status for each healthcare facility, categorized as having or not having an on-site vaccination clinic. Age group was categorized into four groups for the full sample and non-NIP vaccine analyses: 0–17, 18–39, 40–59, and ≥60 years. The NIP vaccine subgroup consisted exclusively of children aged 6 years and younger; thus, age was not analyzed as a predictor in this subgroup. Sex was categorized as male or female.

### 2.3. Statistical Analysis

All statistical analyses were performed using R software (version 4.5.3). Subgroup analyses were stratified by vaccine type (NIP vs. non-NIP). Descriptive statistics were presented as frequencies, percentages, and medians with IQR.

For univariate analyses, categorical variables were compared using the χ^2^ test, and continuous variables using the Mann–Whitney U test or Kruskal–Wallis H test. The principal conversion rates were reported with 95% confidence intervals (CIs) calculated using the Wilson method.

Multivariable analyses comprised two components. For conversion behavior, the analytical strategy varied by subgroup. In the non-NIP subgroup, mixed-effects logistic regression with random intercepts for patient ID was initially considered. However, due to quasi-complete separation caused by extreme differences in conversion rates between manual and electronic prescriptions, the model failed to converge; Firth-corrected logistic regression was therefore applied for this subgroup. In the NIP subgroup, by contrast, mixed-effects models converged for conversion-rate analysis and were used accordingly. For same-day conversion analyses within the NIP subgroup, Firth-corrected logistic regression was used due to the limited number of same-day conversion events and associated convergence issues. For conversion timeliness, a two-part model was used. The first part examined factors associated with same-day conversion using mixed-effects logistic regression with random intercepts for patient ID where convergence was achieved, and Firth-corrected logistic regression where it was not. The second part analyzed log-transformed time to conversion for non-same-day conversions using mixed-effects linear regression with robust standard errors, with results presented as exp(β) and 95% CI. This stratified approach ensured appropriate handling of model convergence across different subgroup analyses.

Interaction terms were included to test effect modification by age or vaccine type. In the non-NIP subsample, age × prescription format and age × vaccination clinic availability were included. In the full sample, only prescription format × vaccine type was included, as only seven NIP prescriptions were issued at facilities with vaccination clinics. Interaction significance was assessed using Type III Wald χ^2^ tests, nested model F-tests, or likelihood ratio tests. When *p* < 0.05, simple effects were estimated via EM Means pairwise comparisons. *p*-values for interaction tests and pairwise comparisons were adjusted using the Holm method.

To account for potential clustering at the healthcare facility level, we conducted a sensitivity analysis using generalized estimating equations (GEE) with healthcare facility as the clustering variable and cluster-robust standard errors. The GEE estimates were directionally consistent with those from the primary Firth-corrected logistic regression model, with all OR differences < 20%, indicating that facility-level clustering did not materially affect the core findings (Supplementary Table S1).

### 2.4. Ethical Approval

This retrospective study was conducted in accordance with the Declaration of Helsinki. As the study involved secondary analysis of routinely collected administrative data from 2025 with no prospective data collection or interventions, ethical review was conducted after study completion as a retrospective exemption review. The study was approved by the Ethics Review Committee of the Henan Provincial Center for Disease Control and Prevention (Approval No.: 2026-KY-038-01; Date of Approval: 30 June 2026). All data were de-identified prior to analysis, and patient confidentiality was strictly protected throughout the research process.

## 3. Results

### 3.1. Baseline Characteristics of the Study Population

A total of 50,887 vaccine prescriptions were included in this study, with an overall conversion rate of 13.8% (95% CI: 13.5–14.1%; 7043/50,887). The study population had a roughly balanced sex distribution (47.2% male vs. 52.8% female), with a median age of 67 years (IQR: 16–73).

Among all prescriptions, 98.0% (49,864/50,887) were non-NIP vaccines, while NIP vaccines accounted for 2.0% (1023/50,887). Electronic prescriptions made up 95.9% (48,813/50,887) of the total, and manual prescriptions accounted for 4.1% (2074/50,887). Prescriptions issued at facilities with an on-site vaccination clinic represented 1.9% (974/50,887) of the total.

Among the converted prescriptions, 54.4% (3831/7043) were vaccinated on the same day the prescription was issued. For non-same-day conversions, the median time to conversion was 6 days (IQR: 2–50).

The baseline characteristics of the study population are summarized in Table 1.

### 3.2. NIP Vaccine Subgroup Analysis

#### 3.2.1. Overall Conversion

A total of 1023 NIP vaccine prescriptions were included, with a conversion rate of 76.8% (95% CI: 74.2–79.3%; 786/1023). A total of 13.9% (95% CI: 11.8–16.2%; 142/1023) were vaccinated on the same day, and 70.0% (644/1023) were non-same-day conversions. The median time to conversion for non-same-day conversions was 23 days (IQR: 6–69).

The NIP vaccine subgroup comprised exclusively children aged 6 years and younger, with a narrow age range and vaccination decisions made by guardians. Additionally, only seven prescriptions in this subgroup were issued at facilities with vaccination clinics. Therefore, age, sex, and vaccination clinic availability were not included as predictors in the analyses for this subgroup.

After excluding vaccines with a frequency of <10, eight vaccines remained for analysis, comprising 1013 valid cases. Fisher‘s exact test revealed a statistically significant difference in conversion rates across vaccine types (*p* < 0.001), with all vaccines achieving conversion rates exceeding 60%. The DTaP vaccine had the highest conversion rate (90.2%, 119/132), while the hepatitis A vaccine had the lowest (62.2%, 46/74). The difference in same-day conversion rates was also significant (Fisher’s exact test, *p* < 0.001), with the hepatitis A vaccine showing the highest same-day conversion rate (28.4%,21/74), and the Polio vaccine (10.0%,15/150) and the MMR vaccine (10.0%,6/60) the lowest. The Kruskal–Wallis test showed a significant difference in non-same-day conversion timeliness across vaccines (H = 36.791, *p* < 0.001), with the Meningococcal polysaccharide vaccine (Groups A+C) having the longest median time to conversion (52 days, IQR: 7–163) and the MMR vaccine the shortest (7 days, IQR: 2–18).

Table 2 presents a detailed comparison of conversion indicators across NIP vaccine types.

#### 3.2.2. Impact of Prescription Format on Conversion Rate and Conversion Timeliness

In univariate analyses, manual prescriptions had a significantly higher conversion rate than electronic prescriptions (92.0% vs. 75.4%; χ^2^ = 11.304, *p* = 0.001), a significantly higher same-day conversion rate (46.0% vs. 10.9%; χ^2^ = 58.981, *p* < 0.001), and significantly shorter time to conversion for non-same-day conversions (W = 14,654.000, *p* = 0.024). After accounting for within-individual correlation using mixed-effects models, manual prescriptions remained significantly associated with higher conversion odds (OR = 3.502, 95% CI: 1.742–8.186, *p* < 0.001). The effect on same-day conversion showed a positive trend but did not reach statistical significance (OR = 2.353, 95% CI: 0.248–22.29, *p* = 0.456), possibly due to the limited number of same-day conversion events. Mixed-effects linear regression showed that manual prescriptions significantly shortened time to conversion for non-same-day conversions (exp(β) = 0.558, 95% CI: 0.359–0.867, *p* = 0.010).

### 3.3. Non-NIP Vaccine Subgroup Analysis

#### 3.3.1. Overall Conversion

A total of 49,864 non-NIP vaccine prescriptions were included, with a conversion rate of 12.5% (95% CI: 12.3–12.8%; 6257/49,864). A total of 7.4% (95% CI: 7.2–7.6%; 3689/49,864) were vaccinated on the same day. The median time to conversion for non-same-day conversions was 4 days (IQR: 1–43). Cumulative conversion rates at 7, 30, and 90 days among delayed converters are presented in Supplementary Table S3.

After excluding vaccines with a frequency of <10, 18 vaccines remained for analysis, comprising 49,839 valid cases. Fisher‘s exact test revealed a statistically significant difference in conversion rates across vaccine types (*p* < 0.001). The EV71 vaccine had the highest conversion rate (82.9%, 63/76), followed by the rotavirus vaccine (81.8%, 27/33). The same-day conversion rates also differed significantly across vaccines (Fisher‘s exact test, *p* < 0.001), with the EV71 vaccine showing the highest rate (60.5%,46/76), followed by the Rotavirus vaccine (51.5%,17/33) and the Rabies vaccine (50.4%,333/661). The Kruskal–Wallis test revealed a significant difference in non-same-day conversion timeliness across vaccines (H = 185.642, *p* < 0.001). The Meningococcal polysaccharide vaccine (Groups A+C) had the longest median time to conversion (102 days; IQR not applicable due to small sample size), while the Rabies and Japanese encephalitis vaccines had the shortest (1 day; IQR: 1–3 for both).

In sensitivity analysis excluding rabies vaccines (*n* = 661), the overall conversion rate for non-NIP vaccines changed from 12.5% to 11.9%, and the same-day conversion rate from 7.4% to 6.8%, indicating that the inclusion of rabies vaccines did not materially affect the main conclusions.

Table 3 presents a detailed comparison of conversion indicators across non-NIP vaccine types.

#### 3.3.2. Univariate Analysis of Factors Associated with Conversion Rate and Conversion Timeliness

Univariate analysis revealed that prescription format (χ^2^ = 9593.062, *p* < 0.001), vaccination clinic availability (χ^2^ = 2660.512, *p* < 0.001), and age group (χ^2^ = 7165.910, *p* < 0.001) all had significant associations with conversion rate, while sex did not (χ^2^ = 0.116, *p* = 0.733). The conversion rate for manual prescriptions was significantly higher than that for electronic prescriptions (83.9% vs. 9.6%). Prescriptions issued at facilities with a vaccination clinic had a significantly higher conversion rate than those issued at facilities without a clinic (67.0% vs. 11.5%). Across age groups, the 0–17 years group had the highest conversion rate (33.8%, 4061/12,020), while the ≥60 years group had the lowest (4.0%, 1299/32,325).

Stratified by age group, significant differences in conversion rates were observed for specific vaccines. For influenza vaccine (χ^2^ = 2090.372, *p* < 0.001), pneumococcal vaccine (χ^2^ = 700.318, *p* < 0.001), and hepatitis B vaccine (χ^2^ = 111.445, *p* < 0.001), the 0–17 years group had the highest conversion rates (28.5%, 1340/4702; 12.3%, 178/1451; and 45.1%, 610/1353, respectively), while the ≥60 years group had the lowest (5.9%, 1114/18,831; 0.8%, 72/9187; and 12.5%, 15/120, respectively). For herpes zoster vaccine (χ^2^ = 200.442, *p* < 0.001), the 40–59 years group had the highest conversion rate (9.2%, 24/262), followed by the ≥60 years group (0.5%, 18/3829). For HPV vaccine (for individuals aged <45 years; χ^2^ = 80.635, *p* < 0.001), the 40–45 years group (>45 years excluded) had the highest conversion rate (36.1%, 57/158), followed by the 18–39 years group (20.3%, 146/719).

Prescription format (χ^2^ = 8061.917, *p* < 0.001), vaccination clinic availability (χ^2^ = 4878.568, *p* < 0.001), and age group (χ^2^ = 4430.654, *p* < 0.001) all significantly associated with the same-day conversion rate. The same-day conversion rate for manual prescriptions was significantly higher than that for electronic prescriptions (59.1%vs. 5.3%). Prescriptions issued at facilities with a vaccination clinic had a significantly higher same-day conversion rate than those issued at facilities without a clinic (65.7%vs. 6.2%). Across age groups, the 0–17 years group had the highest same-day conversion rate (20.1%,2417/12,020), while the ≥60 years group had the lowest (1.9%,615/32,325). Sex did not have a significant effect (χ^2^ = 0.809, *p* = 0.369).

Among non-same-day conversions, prescription format (Z = −16.631, *p* < 0.001) and age group (H = 106.574, *p* < 0.001) were significantly associated with time to conversion. The median time to conversion for manual prescriptions was significantly shorter than that for electronic prescriptions (1 day, IQR: 1–3 vs. 7 days, IQR: 2–72). The 0–17 years (3 days, IQR: 1–39), 18–39 years (2 days, IQR: 1–3), and 40–59 years (2 days, IQR: 1–7) groups had significantly shorter times to conversion than the ≥60 years group (17 days, IQR: 2–65). Vaccination clinic availability (Z = −1.298, *p* = 0.194) and sex (Z = −0.501, *p* = 0.616) did not have significant effects.

Table 4 summarizes the univariate analysis results.

#### 3.3.3. Multivariable Analysis of Factors Associated with Conversion Rate

Multivariable analysis using Firth-corrected logistic regression was performed with conversion status as the dependent variable (reference: not converted), and prescription format, vaccination clinic availability, and age group as independent variables. The results showed that manual prescriptions were associated with 37.720-fold higher odds of conversion compared with electronic prescriptions (95% CI: 32.999–43.180, *p* < 0.001). The presence of a vaccination clinic was associated with 19.386-fold higher odds of conversion compared with no clinic (95% CI: 16.468–22.836, *p* < 0.001). Across age groups, the 0–17 years group (OR = 11.330, 95% CI: 10.519–12.204, *p* < 0.001), the 18–39 years group (OR = 2.884, 95% CI: 2.480–3.354, *p* < 0.001), and the 40–59 years group (OR = 3.901, 95% CI: 3.438–4.425, *p* < 0.001) all had significantly higher conversion odds than the ≥60 years group.

Table 5 summarizes the multivariable analysis results.

#### 3.3.4. Two-Part Model Analysis of Factors Associated with Conversion Timeliness

In the first part, multivariable logistic regression with Firth correction was performed with same-day conversion as the dependent variable (reference: non-same-day conversion), and prescription format, vaccination clinic availability, and age group as independent variables. The results showed that manual prescriptions were associated with 16.119-fold higher odds of same-day conversion compared with electronic prescriptions (95% CI: 14.445–17.999, *p* < 0.001). The presence of a vaccination clinic was associated with 40.303-fold higher odds of same-day conversion compared with no clinic (95% CI: 34.137–47.670, *p* < 0.001). Across age groups, the 18–39 years group had a 4.932-fold higher odds of same-day conversion than the ≥60 years group (95% CI: 4.141–5.859, *p* < 0.001), the 40–59 years group had a 5.890-fold higher odds (95% CI: 5.047–6.865, *p* < 0.001), and the 0–17 years group had a 11.477-fold higher odds (95% CI: 10.359–12.737, *p* < 0.001).

In the second part, mixed-effects linear regression with a random intercept for patient ID was performed with log-transformed time to conversion for non-same-day conversions as the dependent variable, and prescription format and age group as independent variables. The results showed that manual prescriptions significantly shortened the time to conversion for non-same-day conversions, with the time being 0.197 times that of electronic prescriptions (95% CI: 0.166–0.235, *p* < 0.001), corresponding to a reduction of approximately 80.3%. Using the ≥60 years group as the reference, the 0–17 years group (exp(β) = 0.535, 95% CI: 0.453–0.633, *p* < 0.001), the 18–39 years group (exp(β) = 0.259, 95% CI: 0.170–0.394, *p* < 0.001), and the 40–59 years group (exp(β) = 0.286, 95% CI: 0.204–0.400, *p* < 0.001) all had significantly shorter times to conversion, indicating that younger age groups converted faster than the ≥60 years group.

#### 3.3.5. Interaction Analyses of Age with Prescription Format and Vaccination Clinic Availability

In the conversion rate model, the age × prescription format interaction was statistically significant (χ^2^ = 232.964, *p* < 0.001). Manual prescriptions significantly associated with higher conversion across all age groups, but the effect magnitude varied by age. Predicted probability plots for the conversion rate interaction effects are provided in Supplementary Figure S1. The ≥60 years group showed the strongest effect (OR = 166.310, 95% CI: 126.786–218.154), while the 0–17 years group (OR = 19.757, 95% CI: 16.453–23.724), the 18–39 years group (OR = 12.759, 95% CI: 9.115–17.859), and the 40–59 years group (OR = 23.931, 95% CI: 17.212–33.273) all had substantially lower effects. The age × vaccination clinic availability interaction was not statistically significant (χ^2^ = 3.293, *p* = 0.349), suggesting that the effect of vaccination clinics was relatively consistent across age groups.

For same-day conversion, both the age × prescription format (F = 91.423, *p* < 0.001) and age × vaccination clinic availability (F = 4.588, *p* = 0.003) interactions were statistically significant. Predicted probability plots for the same-day conversion interaction effects are presented in Supplementary Figure S2. Manual prescriptions had the strongest effect on improving same-day conversion in the ≥60 years group (ΔP = 0.800, 95% CI: 0.773–0.827), while vaccination clinics were associated with the largest improvement in same-day conversion in the 18–39 years group (ΔP = 0.676, 95% CI: 0.617–0.735), followed by the 40–59 years group (ΔP = 0.672, 95% CI: 0.627–0.718), the 0–17 years group (ΔP = 0.614, 95% CI: 0.598–0.631), and the ≥60 years group (ΔP = 0.595, 95% CI: 0.545–0.646).

For non-same-day conversion timeliness, the age × prescription format interaction was statistically significant (F = 124.712, *p* = 0.002). Manual prescriptions shortened the time to conversion across all age groups, with the strongest effect observed in the ≥60 years group (exp(β) = 0.130, 95% CI: 0.091–0.186). Forest plots for the non-same-day conversion timeliness interaction effects are shown in Supplementary Figure S3.

Table 6 summarizes the interaction analysis results.

#### 3.3.6. Stratified Analysis by Vaccine Category

To examine heterogeneity across non-NIP vaccines, we performed stratified analyses by vaccine category using multivariable logistic regression with adjustment for prescription format, vaccination clinic availability, and age group. Among the 12 vaccines meeting the inclusion criteria (*n* ≥ 30, ≥5 events, and sufficient exposure variation), the direction of associations was broadly consistent with the main analysis, whereas the magnitude varied appreciably across vaccines (Supplementary Table S2). Manual prescriptions showed the strongest associations with herpes zoster (OR = 108.54, 95% CI: 33.95–360.79) and pneumococcal (OR = 82.52, 95% CI: 43.30–162.50) vaccines, whereas weaker associations were observed for rabies (OR = 8.49, 95% CI: 5.17–14.62) and hepatitis A (OR = 2.02, 95% CI: 0.63–8.38). Vaccination clinic availability exhibited the largest effects for herpes zoster (OR = 33.77, 95% CI: 6.32–142.04) and varicella (OR = 26.20, 95% CI: 8.94–126.96). Age effects were consistent with the main findings: the 0–17 years group had substantially higher conversion odds than the ≥60 years group for most vaccines, particularly for hepatitis A (OR = 47.33, 95% CI: 16.11–229.71) and pneumococcal (OR = 18.38, 95% CI: 13.70–24.93) vaccines.

### 3.4. Comparison of Conversion Indicators Between NIP and Non-NIP Vaccines

#### 3.4.1. Comparison of Conversion Rates and Conversion Timeliness Between the Two Vaccine Types

The overall conversion rate for NIP vaccines (76.8%, 786/1023; 95% CI: 74.2–79.3%) was significantly higher than that for non-NIP vaccines (12.5%, 6257/49,864; 95% CI: 12.3–12.8%; χ^2^ = 3473.899, *p* < 0.001). The same-day conversion rate for NIP vaccines (13.9%, 142/1023; 95% CI: 11.9–16.1%) was also significantly higher than that for non-NIP vaccines (7.4%, 3689/49,864; 95% CI: 7.2–7.6%; χ^2^ = 59.585, *p* < 0.001). Among non-same-day conversions, the median time to conversion for NIP vaccines was 23 days (IQR: 6–69), compared with 4 days (IQR: 1–43) for non-NIP vaccines, with the difference being statistically significant (Z = −11.342, *p* < 0.001).

#### 3.4.2. Interaction Between Prescription Format and Vaccine Type

The overall interaction tests were all statistically significant (conversion rate: χ^2^ = 22.275, *p* < 0.001; same-day conversion rate: F = 28.457, *p* < 0.001; non-same-day conversion timeliness: F = 59.937, *p* < 0.001), indicating that vaccine type significantly modified the effects of prescription format across all three outcomes. For conversion rate, the association of manual prescriptions was substantially greater for non-NIP vaccines (OR = 49.128, 95% CI: 43.424–55.582) than for NIP vaccines (OR = 3.723, 95% CI: 1.695–8.177). For same-day conversion rate, manual prescriptions showed a more pronounced improvement in non-NIP vaccines (ΔP = 0.538, 95% CI: 0.516–0.560) than in NIP vaccines (ΔP = 0.351, 95% CI: 0.238–0.464). For non-same-day conversion timeliness, manual prescriptions had a stronger shortening effect on non-NIP vaccines (exp(β) = 0.198, 95% CI: 0.166–0.237) than on NIP vaccines (exp(β) = 0.532, 95% CI: 0.298–0.949).

Table 7 summarizes the interaction analysis results.

## 4. Discussion

This study found that NIP vaccine prescriptions had a substantially higher overall conversion rate than non-NIP vaccines (76.8% vs. 12.5%; χ^2^ = 3473.899, *p* < 0.001), and a higher same-day conversion proportion (13.9% vs. 7.4%; χ^2^ = 59.585, *p* < 0.001). NIP vaccines are required under the NIP schedule with guardian obligations [16,17], and were associated with a strong willingness to complete vaccination [6]. In contrast, non-NIP vaccines are voluntary and self-paid, involving longer decision-making and greater vaccine hesitancy, and were associated with lower final conversion rates [18]. However, vaccine hesitancy alone does not fully account for the low uptake of non-NIP vaccines. A range of additional factors have been identified across individual, provider, and policy levels. At the individual and family level, high out-of-pocket costs remain a major barrier to equitable vaccine uptake, particularly for low-income households [19]; Limited health literacy and lack of awareness about vaccine-preventable diseases further reduce demand [20]. At the provider and system level, inadequate physician–caregiver communication, insufficient provider recommendation, and weak incentives for vaccinators also hinder uptake [21,22,23]. Furthermore, the integration of non-NIP vaccines into routine vaccination services remains incomplete, with suboptimal and inequitable coverage across regions [19]. At the policy level, although some local governments have introduced free vaccination programs for selected non-NIP vaccines, these initiatives face challenges related to sustainability and regional disparities [19].

Prescription format was also identified as an important moderating factor. Manual prescriptions were associated with significantly higher prescription-to-vaccination conversion and timeliness than electronic prescriptions across both vaccine types, with a more pronounced difference for non-NIP vaccines. Manual prescriptions are primarily issued by village doctors or community health centers, and were likely accompanied by physician–patient familiarity and health education and risk communication. Healthcare provider recommendations are a key factor associated with vaccination uptake [24]. As a tangible carrier, manual prescriptions serve as a repeated reminder, reducing attrition due to forgetfulness or procedural complexity. Electronic prescriptions, predominantly issued by large hospitals with more distant physician–patient relationships, lack a physical form and are more susceptible to being forgotten or lost. Previous studies have confirmed that patient reminder and recall interventions are associated with improved vaccination rates [25], high-quality provider recommendations are significantly associated with HPV vaccination [26], and electronic reminders alone, without face-to-face communication, are insufficient to improve influenza vaccination [27]. Thus, while implementing electronic prescriptions, the integration of communication and trust mechanisms from manual prescriptions—including vaccine education and real-time reminders—may be worth considering.

The availability of vaccination clinics also was significantly associated with prescription conversion. In the non-NIP subgroup, facilities with a clinic had a significantly higher same-day conversion odds than those without (OR = 40.303, 95% CI: 34.137–47.670, *p* < 0.001). This finding is consistent with Jacobson Vann et al. (2018), who found that reminder and recall interventions effectively improve vaccination rates (RR = 1.28, 95% CI: 1.23–1.35), and that integrating recommendations and vaccination at the same site reduces patient attrition [25]. An analysis of 42,282 clinics across China demonstrated that clinics with ≥5 vaccination stations (OR = 1.32) and ≥9 staff members (OR = 1.15) had lower DTaP dropout rates [28]. On-site clinics allow for same-location prescription and vaccination, which may reduce cross-institutional attrition. Digitalization also has been associated with positive outcomes: intelligent vaccination clinics in Qingdao were well-rated for improving service efficiency [29], and mobile applications significantly improved vaccination coverage among migrant children [30]. Interaction analyses revealed that on-site vaccination clinics significantly improved same-day conversion across all age groups, with the magnitude of the association being lowest among individuals aged ≥60 years (ΔP = 0.595), whereas it ranged from 0.614 to 0.676 among younger age groups. This may reflect additional barriers faced by older adults, including transportation difficulties, reliance on caregiver assistance, financial constraints, and lower health literacy, which clinic availability alone cannot address. Although the clinic‘s effect on overall conversion did not differ by age, the smaller gain in same-day conversion among older adults suggests that while on-site clinics remove a critical access barrier, they do not fully resolve the multidimensional obstacles delaying vaccination in this population. Future efforts should integrate complementary strategies, such as transportation support and targeted health education, alongside clinic-based interventions to maximize the benefit for older adults.

Older adults (≥60 years) exhibited lower prescription-to-vaccination conversion and timeliness, associated with price sensitivity, limited income, low awareness, and limited accessibility. Real-world data from Shanghai’s Pudong New Area showed that, among individuals aged ≥ 60 years, vaccination coverage during 2020–2024 was 3.12% for influenza, 1.61% for 23-valent pneumococcal polysaccharide, and 0.23% for recombinant herpes zoster vaccines [31]. A survey in a southwest Chinese city found that 55.6% of unvaccinated older adults “did not know or had never heard of” the influenza vaccine [32]; a Jiaozuo survey reported that 43.33% of unvaccinated older adults cited “good health, no need” as their reason for non-vaccination [33]. Vaccine price was identified as a key factor influencing vaccination decisions [34] and was also confirmed as a main reason for non-vaccination among older adults in Weifang City [35]. Limited mobility, distance to vaccination sites, and transportation difficulties significantly reduce access to vaccination services [36,37], with transportation identified as a key barrier in rural Shanxi [38]. Manual prescriptions were substantially associated with higher vaccination efficiency in older adults, with a greater effect than in other age groups, as physicians leverage familiarity and trust to deliver face-to-face health education and risk communication. Family physician recommendations were significantly associated with influenza and pneumococcal vaccination among urban older adults [39]. The strong effect of manual prescriptions among older adults (OR = 166.31) may be attributed to their integration of face-to-face communication, physician trust, and tangible reminders—elements that are particularly critical for this population, given their lower health literacy and greater reliance on interpersonal interactions. In contrast, the relatively modest effect of vaccination clinics on older adults suggests that while on-site clinics reduce cross-institutional attrition, they do not adequately address broader obstacles, including transportation difficulties, financial constraints, and caregiver dependence, which remain key barriers to timely vaccination in this age group.

Future digital health interventions, including AI-assisted vaccine recommendation systems [40], electronic reminder platforms, automated scheduling [41], and clinical decision support integrated within electronic medical records, hold promise for further enhancing vaccine prescription conversion. These tools may streamline workflows by standardizing risk assessment and personalizing vaccine recommendations, reduce patient attrition through automated reminders and scheduling, and enable closed-loop management from prescription to vaccination through real-time data interoperability. Notably, the optimal role of digital interventions is not to replace the communication and trust-building functions of manual prescriptions, but rather to complement them, particularly in primary-care settings where digital tools can manage routine workflows at scale, while manual prescriptions can be reserved for high-risk or vaccine-hesitant populations requiring enhanced interpersonal communication. This combined approach warrants further investigation in future implementation studies.

This study has several limitations. First, the data were derived exclusively from a single county-level pilot city (Lingbao), limiting generalizability to regions with different healthcare systems, though the findings remain reasonably representative within Henan Province. Second, the small number of NIP vaccine prescriptions issued at facilities with vaccination clinics (n = 7) precluded stable estimation of the vaccine type × vaccination clinic interaction. Third, as a retrospective observational study using administrative data, unequal follow-up periods (ranging from 4 to 16 months) due to the unified cutoff date; key individual- and provider-level confounders (e.g., comorbidities, socioeconomic status, health literacy, and physician characteristics) were unavailable; and manual prescriptions were predominantly issued in primary-care settings where higher-risk patients were more likely to receive them, potentially introducing selection bias. Accordingly, the large odds ratios should be interpreted as composite associations reflecting a bundle of service-delivery characteristics (manual format, enhanced communication, patient trust, and primary-care setting) rather than causal effects. Patient-level factors that may explain the low conversion rates among older adults (e.g., transportation barriers, vaccine hesitancy, affordability, and caregiver assistance) were not measured and warrant future prospective investigation.

## 5. Conclusions

In summary, the overall vaccine prescription conversion rate was low, with significant differences between NIP and non-NIP vaccines. Manual prescriptions and vaccination clinics were the two factors most strongly associated with conversion. Age significantly modified the associations of prescription format and clinic availability, and the ≥60 years group showed the strongest associations with these factors. Based on these findings, it may be prudent to consider retaining manual prescriptions as a supplement to electronic systems, particularly for non-NIP vaccines and the ≥60 years population. Priority may be given to establishing vaccination clinics at prescribing facilities to reduce cross-institutional attrition. An age-stratified approach could be considered, with enhanced face-to-face communication for the ≥60 years group and greater emphasis on appointment reminders for children and NIP vaccines.

## Figures and Tables

**Table 1:** Baseline characteristics of the study population (*N* = 50,887).

Variable	Category	*n*/Median	%
Overall conversion	Converted	7043	13.8
	Not converted	43,844	86.2
Vaccine type	NIP	1023	2.0
	Non-NIP	49,864	98.0
Sex	Male	24,015	47.2
	Female	26,872	52.8
Age	Median (IQR)	67 (16–73)	—
Prescription format	Manual	2074	4.1
	Electronic	48,813	95.9
Vaccination clinic	Available	974	1.9
	Not available	49,913	98.1
Conversion type	(Among converted, *n* = 7043)		
	Same- day	3831	54.4
	Non-same day	3212	45.6
Time to conversion	(Among non-same day)		
	Median days (IQR)	6 (2–50)	—

Data are presented as *n* (%), median (IQR), or as indicated. NIP: National Immunization Program; IQR: interquartile range; —: not applicable.

**Table 2:** Comparison of conversion indicators across NIP vaccine types (*N* = 1013).

Vaccine Type	Total (*n*)	Conversion Rate (%)	Same-Day Conversion Rate (%)	Median Time to Conversion for Non-Same-Day (Days, IQR)
DTaP	132	90.2 (119/132)	13.6 (18/132)	15 (4–43)
HepB	203	82.3 (167/203)	16.7 (34/203)	23 (10–58)
JEV	81	80.2 (65/81)	13.6 (11/81)	19 (9–53)
MenA	68	77.9 (53/68)	13.2 (9/68)	40 (7–97)
Polio	150	76.0 (114/150)	10.0 (15/150)	17 (4–49)
MenAC	245	74.3 (182/245)	11.4 (28/245)	52 (7–153)
HepA	74	62.2 (46/74)	28.4 (21/74)	23 (8–55)
MMR	60	63.3 (38/60)	10.0(6/60)	8 (2–18)
Total	1013	77.4 (784/1013)	14.0(142/1013)	23 (6–69)
Test statistic		Fisher’s exact test	Fisher’s exact test	H = 36.791
*p*-value		<0.001	<0.001	<0.001

Data are *n*/*N* (converted cases/total sample). IQR: interquartile range. χ^2^ tests were used where expected frequencies were ≥5; otherwise, Fisher‘s exact test (with simulated *p*-value) was applied. Abbreviations: DTaP, diphtheria–tetanus–acellular pertussis; HepB, hepatitis B; JEV, Japanese encephalitis; MenA, meningococcal polysaccharide (Group A); MenAC, meningococcal polysaccharide (Groups A+C); Polio, poliovirus; HepA, hepatitis A; MMR, measles–mumps–rubella.

**Table 3:** Comparison of conversion indicators across non-NIP vaccine types (*N* = 49,839).

Vaccine Type	Total (*n*)	Conversion Rate (%)	Same-Day Conversion Rate (%)	Median Time to Conversion for Non-Same-Day (Days, IQR)
EV71	76	82.9 (63/76)	60.5 (46/76)	2 (1–5)
RV	33	81.8 (27/33)	51.5 (17/33)	2 (1–2)
JEV	56	76.8 (43/56)	47.5 (28/56)	1 (1–3)
DTaP	56	76.8 (43/56)	12.5 (7/56)	7 (1–20)
Hib	38	76.3 (29/38)	50.0 (19/38)	2 (1–12)
Rabies	661	60.4 (399/661)	50.4 (333/661)	1 (1–3)
MenACWY	1169	56.9 (665/1169)	32.3 (378/1169)	3 (1–30)
Varicella	933	54.1 (505/933)	26.7 (249/933)	2 (1–4)
HepA	659	52.0 (343/659)	33.1 (218/659)	3 (1–18)
HepB	1807	38.2 (690/1807)	27.0 (488/1807)	3 (1–29)
MenAC	34	23.5 (8/34)	20.6 (7/34)	102 (—)
HPV	1446	17.1 (247/1446)	9.8 (174/1446)	2 (1–22)
Influenza	26,159	10.9 (2856/26,159)	5.9 (1539/26,159)	12 (2–72)
Pneumococcal	11,532	2.5 (283/11,532)	1.4 (159/11,532)	5 (2–46)
Tetanus	355	1.4 (5/355)	0.6 (2/355)	25 (4–—)
HZV	4425	0.9 (42/4425)	0.5 (21/4425)	12 (1–66)
HFRS	204	0.0 (0/204)	—	—
HepE	196	0.0 (0/196)	—	—
Total	49,839	12.5 (6248/49,839)	7.4 (3685/49,839)	4 (1–43)
Test statistic		Fisher‘s exact test	Fisher‘s exact test	*H* = 185.642
*p*-value		<0.001	<0.001	<0.001

Data are *n*/*N* (converted cases/total sample). IQR: interquartile range; —: not applicable. χ^2^ tests were used where expected frequencies were ≥5; otherwise, Fisher‘s exact test (with simulated *p*-value) was applied; Kruskal–Wallis H tests were used for time to conversion. Abbreviations: DTaP, diphtheria–tetanus–acellular pertussis; EV71, enterovirus 71; HepA, hepatitis A; HepB, hepatitis B; HepE, hepatitis E; HFRS, hemorrhagic fever with renal syndrome; Hib, *Haemophilus influenzae* type b; HPV, human papillomavirus; HZV, herpes zoster; JEV, Japanese encephalitis; MenAC, meningococcal polysaccharide (Groups A+C); MenACWY, meningococcal polysaccharide (Groups ACYW135); RV, rotavirus. The non-NIP vaccines listed here include self-paid alternatives to NIP vaccines (e.g., DTaP, hepatitis B, hepatitis A) from different manufacturers or formulations.

**Table 4:** Univariate analysis of factors associated with conversion rate and conversion timeliness in the non-NIP vaccine subgroup.

Outcome Variable	Factor	Category	Conversion Rate (%)	Median (Days, IQR)	Test Statistic	*p*-Value
Conversion rate	Prescription format	Manual	83.9 (1667/1987)	—	χ^2^ = 9593.062	<0.001
		Electronic	9.6 (4590/47,877)	—		
	Vaccination clinic	Available	67.0 (648/967)	—	χ^2^ = 2660.512	<0.001
		Not available	11.5 (5609/48,897)			
	Age group	0–17 years	33.8 (4061/12,020)	—	χ^2^ = 7165.910	<0.001
		18–39 years	14.4 (348/2417)	—		
		40–59 years	17.7 (549/3102)	—		
		≥60 years	4.0 (1299/32,325)	—		
	Sex	Male	12.6 (2958/23,469)	—	χ^2^ = 0.116	0.733
		Female	12.5 (3299/26,395)	—		
Same-day conversion rate	Prescription format	Manual	59.1(1174/1987)	—	χ^2^ = 8061.917	<0.001
		Electronic	5.3(2515/47,877)	—		
	Vaccination clinic	Available	65.7 (635/967)	—	χ^2^ = 4878.568	<0.001
		Not available	6.2(3054/48,897)	—		
	Age group	0–17 years	20.1(2417/12,020)	—	χ^2^ = 4430.654	<0.001
		18–39 years	10.8(261/2417)	—		
		40–59 years	12.8(396/3102)	—		
		≥60 years	1.9(615/32,325)	—		
	Sex	Male	7.5(1763/23,469)	—	χ^2^ = 0.809	0.369
		Female	7.3(1926/26,395)	—		
Time to conversion (non-same-day)	Prescription format	Manual	—	1 (1–3)	Z = −16.631	<0.001
		Electronic	—	7 (2–72)		
	Vaccination clinic	Available	—	1 (1–23)	Z = −1.298	0.194
		Not available	—	4 (1–43)		
	Age group	0–17 years	—	3 (1–39)	H = 106.574	<0.001
		18–39 years	—	2 (1–3)		
		40–59 years	—	2 (1–7)		
		≥60 years	—	17 (2–65)		
	Sex	Male	—	4 (1–49)	Z = −0.501	0.616
		Female	—	3 (3–39)		

Data are *n*/*N* (converted cases/total sample). IQR: interquartile range; —: not applicable. χ^2^ tests were used for conversion rate and same-day conversion rate; Mann–Whitney Z tests and Kruskal–Wallis H tests were used for time to conversion.

**Table 5:** Multivariable analysis of factors associated with conversion rate and conversion timeliness in the non-NIP vaccine subgroup.

Outcome Variable	Predictor	Category (Reference)	Effect Estimate	95% CI	*p*-Value
Conversion rate	Prescription format	Manual (vs. Electronic)	OR = 37.720	32.999–43.180	<0.001
	Vaccination clinic	Available (vs. Not available)	OR = 19.386	16.468–22.836	<0.001
	Age group	0–17 years (vs. ≥60 years)	OR = 11.330	10.519–12.204	<0.001
		18–39 years (vs. ≥60 years)	OR = 2.884	2.480–3.354	<0.001
		40–59 years (vs. ≥60 years)	OR = 3.901	3.438–4.425	<0.001
Same-day conversion rate	Prescription format	Manual (vs. Electronic)	OR = 16.119	14.445–17.999	<0.001
	Vaccination clinic	Available (vs. Not available)	OR = 40.303	34.137–47.670	<0.001
	Age group	0–17 years (vs. ≥60 years)	OR = 11.477	10.359–12.737	<0.001
		18–39 years (vs. ≥60 years)	OR = 4.932	4.141–5.859	<0.001
		40–59 years (vs. ≥60 years)	OR = 5.890	5.047–6.865	<0.001
Time to conversion (non-same-day)	Prescription format	Manual (vs. Electronic)	exp(β) = 0.197	0.166–0.235	<0.001
	Age group	0–17 years (vs. ≥60 years)	exp(β) = 0.535	0.453–0.633	<0.001
		18–39 years (vs. ≥60 years)	exp(β) = 0.259	0.170–0.394	<0.001
		40–59 years (vs. ≥60 years)	exp(β) = 0.286	0.204–0.400	<0.001

OR: odds ratio; exp(β): exponentiated coefficient; CI: confidence interval. All models were adjusted for prescription format, vaccination clinic availability, and age group. The time-to-conversion model was adjusted for prescription format and age group only, as vaccination clinic availability was not significant in univariate analysis (*p* = 0.194). For time to conversion, linear regression was performed on log-transformed values due to positive skewness.

**Table 6:** Interaction effects of age × prescription format and age × vaccination clinic availability on prescription-to-vaccination conversion and timeliness.

Outcome Variable	Interaction Term	Overall Interaction Test		Age Group	Effect Estimate	95% CI	*p*-Value
		Test Statistic	*p*-Value				
Conversion rate (OR)	Age × Prescription format	χ^2^ = 232.964	<0.001	0–17	19.757	16.453–23.724	<0.001
				18–39	12.759	9.115–17.859	<0.001
				40–59	23.931	17.212–33.273	<0.001
				≥60	166.310	126.786–218.154	<0.001
	Age × Vaccination clinic	χ^2^ = 3.293	0.349	0–17	25.727	17.343–38.165	<0.001
				18–39	16.717	10.020–27.890	<0.001
				40–59	23.756	15.053–37.492	<0.001
				≥60	18.459	15.033–22.665	<0.001
Same-day conversion rate (ΔP)	Age × Prescription format	F = 91.423	<0.001	0–17	0.399	0.377–0.421	<0.001
				18–39	0.570	0.519–0.621	<0.001
				40–59	0.567	0.525–0.609	<0.001
				≥60	0.800	0.773–0.827	<0.001
	Age × Vaccination clinic	F = 4.588	0.003	0–17	0.614	0.598–0.631	<0.001
				18–39	0.676	0.617–0.735	<0.001
				40–59	0.672	0.627–0.718	<0.001
				≥60	0.595	0.545–0.646	<0.001
Time to conversion (non-same-day) (exp(β))	Age × Prescription format	F = 124.712	<0.001	0–17	0.214	0.171–0.266	<0.001
				18–39	0.426	0.163–1.111	0.081
				40–59	0.484	0.246–0.951	0.035
				≥60	0.130	0.091–0.186	<0.001

OR: odds ratio; ΔP: change in probability estimated from model-predicted probabilities via estimated marginal means (EM Means); exp(β): exponentiated coefficient from linear regression on log-transformed time to conversion; CI: confidence interval. All models used electronic prescriptions, no vaccination clinic, and ≥60 years as the reference groups. For conversion rate, binary logistic regression was used; for same-day conversion rate, the ΔP was derived from predicted probabilities of the fitted logistic regression models via EM Means; for time to conversion, linear regression was performed on log-transformed values due to positive skewness.

**Table 7:** Interaction effects of prescription format × vaccine type on prescription-to-vaccination conversion and timeliness.

Outcome Variable	Interaction Test	*p*-Value	Vaccine Type	Effect Estimate	95% CI	*p*-Value
Conversion rate (OR)	χ^2^ = 22.275	<0.001	NIP	3.723	1.695–8.177	0.001
			Non-NIP	49.128	43.424–55.582	<0.001
Same-day conversion rate (ΔP)	F = 28.457	<0.001	NIP	0.351	0.238–0.464	<0.001
			Non-NIP	0.538	0.516–0.560	<0.001
Time to conversion (non-same-day) (exp(β))	F = 59.937	<0.001	NIP	0.532	0.298–0.949	0.033
			Non-NIP	0.198	0.166–0.237	<0.001

OR: odds ratio; ΔP: change in probability estimated from model-predicted probabilities via estimated marginal means (EM Means); exp(β): exponentiated coefficient from linear regression on log-transformed time to conversion; CI: confidence interval. All models used NIP vaccines and electronic prescriptions as the reference groups. For conversion rate, binary logistic regression was used; for same-day conversion rate, the ΔP was derived from predicted probabilities of the fitted logistic regression models via EM Means; for time to conversion, linear regression was performed on log-transformed values due to positive skewness.

## Data Availability

Restrictions apply to the availability of these data. Data were obtained from the Henan Provincial Immunization Information Management System and healthcare facilities in Lingbao City, and are available from the corresponding author upon reasonable request and with the permission of the Henan Provincial Center for Disease Control and Prevention.

## Supplementary Materials

The following supporting information can be downloaded at: https://www.mdpi.com/article/10.3390/vaccines14080720/s1, Table S1: Sensitivity analysis for facility-level clustering (GEE); Table S2: Stratified analysis by non-NIP vaccine category: multivariable logistic regression results adjusting for prescription format, vaccination clinic availability, and age group; Table S3: Kaplan–Meier cumulative conversion rates at 7, 30, and 90 days among delayed converters (non-same-day conversions), by prescription format and age group; Figure S1: Predicted conversion probability: interaction effects; Figure S2: Predicted same-day conversion probability: interaction effects; Figure S3: Non-same-day conversion timeliness: interaction effects.

## Abbreviations

The following abbreviations are used in this manuscript:

NIP	National Immunization Program
HIS	Hospital Information System
IQR	interquartile range
OR	odds ratio
CI	confidence interval
DTaP	diphtheria–tetanus–acellular pertussis
EV71	enterovirus 71
HepA	hepatitis A
HepB	hepatitis B
HepE	hepatitis E
HFRS	hemorrhagic fever with renal syndrome
Hib	Haemophilus influenzae type b
HPV	human papillomavirus
HZV	herpes zoster
JEV	Japanese encephalitis
MenA	meningococcal polysaccharide (Group A)
MenAC	meningococcal polysaccharide (Groups A+C)
MenACWY	meningococcal polysaccharide (Groups ACYW135)
MMR	measles–mumps–rubella
RV	rotavirus
