# Supplementary material for: Factors Associated with Prescription-to-Vaccination Conversion and Timeliness: A Real-World Analysis
Source: Vaccines (Basel). 2026 Aug 20;14(8):720. doi: 10.3390/vaccines14080720 (PMC13517509; doi:10.3390/vaccines14080720)
Supplement: Supplementary file 1 [file vaccines-14-00720-s001.zip › vaccines-4490781-supplementary.pdf]

Supplementary Table S1. Sensitivity analysis for facility-level clustering (GEE)

Variable	Primary Firth-corrected OR (95% CI)	GEE with facility clustering OR (95% CI)
Manual prescription (vs. electronic)	37.720 (32.999–43.180)	30.546 (26.028–35.848)
Vaccination clinic available (vs. not)	19.386 (16.468–22.836)	18.603 (15.538–22.273)
Age 0–17 (vs. ≥60)	11.330 (10.519–12.204)	12.299 (11.355–13.320)
Age 18–39 (vs. ≥60)	2.884 (2.480–3.354)	3.049 (2.598–3.579)
Age 40–59 (vs. ≥60)	3.901 (3.438–4.425)	4.119 (3.605–4.706)

Table S1 footnotes: OR: odds ratio; CI: confidence interval. GEE: generalized estimating equations. The GEE model used healthcare facility as the clustering variable with an exchangeable correlation structure.

Supplementary Table S2. Stratified analysis by non-NIP vaccine category: multivariable logistic regression results adjusting for prescription format, vaccination clinic availability, and age group

Vaccine Type	N	Events	Prescription format OR (95% CI)	Vaccination clinic OR(95%CI)	Age group: 0–17 vs. ≥60, OR (95% CI)	Age group: 18–39 vs. ≥60, OR (95% CI)	Age group: 40–59 vs. ≥60, OR (95% CI)
RV	33	27	4.41 (0.75–47.21)	1.43 (0.08–219.06)	—	—	—
EV71	76	63	22.62 (2.75–2946.69)	3.57 (0.34–486.45)	—	0.01 (0–0.23)	—
Tetanus	355	5	21.38 (3.56–149.11)	0.78 (0–18.80)	0.09 (0–1.06)	1.41 (0.18–8.96)	0.21 (0–2.45)
HepA	659	343	2.02 (0.63–8.38)	0.62 (0.05–6.26)	47.33 (16.11-229.71)	3.13 (0.58–19.63)	9.89 (2.40–56.43)
Rabies	661	399	8.49 (5.17–14.62)	9.60 (4.19–26.83)	0.65 (0.39–1.06)	0.60 (0.31–1.17)	1.19 (0.64–2.20)
Varicella	933	505	10.57 (4.75–28.97)	26.20 (8.94–126.96)	2.87 (0.15–418.85)	0.14 (0–31.31)	0.27 (0–61.13)
MenACWY	1,169	665	15.60 (11.32–21.88)	20.77 (2.34–2730.87)	—	0.21 (0.02–1.37)	—
HPV	1,446	247	10.05 (5.79–17.84)	15.53 (6.87–38.13)	—	2.67 (1.85–3.92)	7.00 (4.41–11.21)
HepB	1,807	690	16.22 (10.75–25.20)	5.59 (3.24–10.02)	9.84 (5.17–20.50)	1.41 (0.64–3.25)	1.57 (0.71–3.64)
HZV	4,425	42	108.54 (33.95–360.79)	33.77 (6.32–142.04)	0.02 (0–0.84)	0.57 (0–5.68)	13.62 (6.75–27.35)
Pneumococcal	11,532	283	82.52 (43.30–162.50)	11.15 (5.74–20.45)	18.38 (13.70–24.93)	2.23 (0.60–5.91)	4.15 (2.50–6.65)
Influenza	26,159	2,856	56.41 (46.00–69.66)	24.53 (19.95–30.26)	6.26 (5.67–6.92)	1.34 (1.03–1.73)	3.06 (2.58–3.60)

Table S2 footnotes: CI, confidence interval; EV71, enterovirus 71; HepA, hepatitis A; HepB, hepatitis B; HPV, human papillomavirus; HZV, herpes zoster; MenACWY, meningococcal polysaccharide (Groups ACYW135); OR, odds ratio; RV, rotavirus. Data are presented as OR (95% CI). All models were adjusted for prescription format, vaccination clinic availability, and age group simultaneously. Age groups are compared against the reference group (≥60 years). "—" indicates no observations in that age group. Vaccines excluded from this stratified analysis (e.g., JEV, DTaP, Hib, MenAC, HFRS, HepE) had sample sizes <30, <5 events, or insufficient exposure variation, and together accounted for 0.2% of all non-NIP prescriptions. Wide confidence intervals for some estimates (e.g., EV71, RV) reflect small sample sizes and should be interpreted with caution.

Supplementary Table S3. Kaplan-Meier cumulative conversion rates at 7, 30, and 90 days among delayed converters (non-same-day conversions), by prescription format and age group

Table S3A. By prescription format

Prescription Format	Days	Cumulative Conversion(%)	95% CI	N at risk
Electronic	7	50.0	47.8–52.1	1,058
Electronic	30	64.8	62.7–66.8	739
Electronic	90	78.2	76.4–79.9	459
Manual	7	88.6	85.5–91.1	68
Manual	30	97.2	95.2–98.3	15
Manual	90	99.2	97.9–99.7	4

Table S3B. By age group

Age Group	Days	Cumulative Conversion(%)	95% CI	N at risk
0–17 years	7	61.4	59.0–63.7	654
0–17 years	30	73.0	70.8–75.1	448
0–17 years	90	81.7	79.7–83.5	305
18–39 years	7	77.0	66.2–84.4	20
18–39 years	30	85.1	75.3–91.0	13
18–39 years	90	93.1	85.1–96.8	7
40–59 years	7	76.5	68.7–82.3	39
40–59 years	30	88.2	81.8–92.4	18
40–59 years	90	92.8	87.3–95.9	11
≥60 years	7	41.1	37.3–44.7	413
≥60 years	30	60.5	56.7–64.0	275
≥60 years	90	79.8	76.6–82.6	140

Table S3 footnotes: Data are presented as KM-estimated cumulative conversion rates (95% CI). The analysis was restricted to delayed converters (non-same-day conversions); non-converters were censored at April 26, 2026. CI, confidence interval; KM, Kaplan-Meier.

Supplementary Figure S1. Predicted conversion probability: interaction effects.

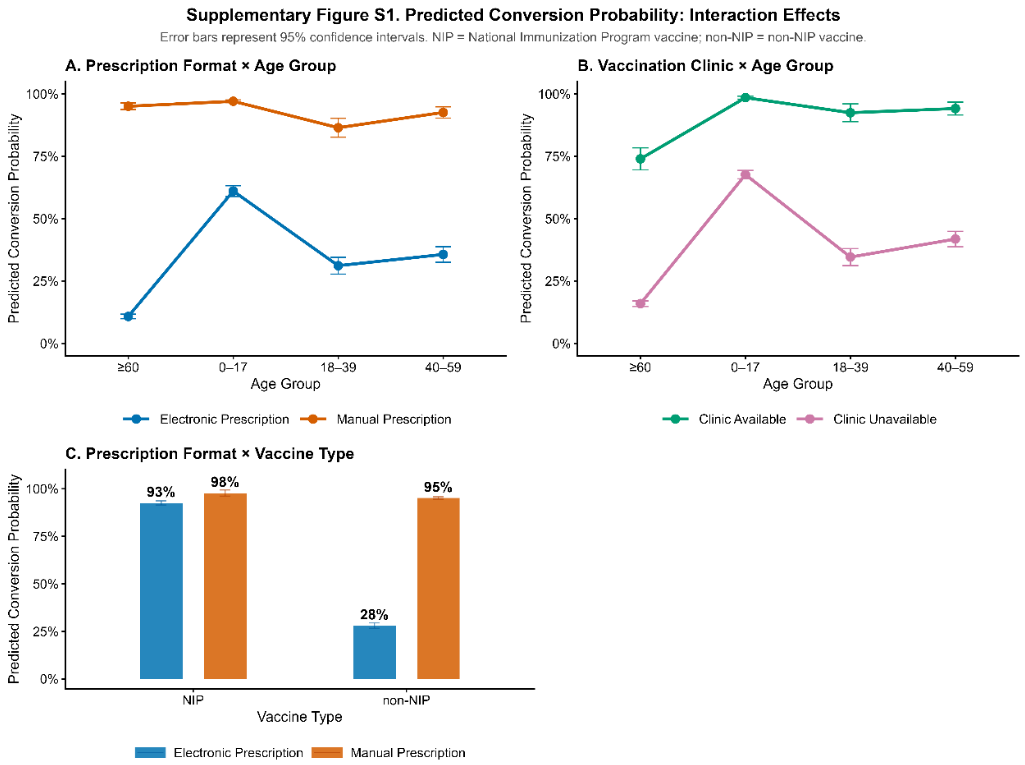

Figure S1 footnotes: Panels show predicted probabilities (with 95% CIs) derived from estimated marginal means (EM Means) based on Firth-corrected logistic regression models. (A) predicted probability by prescription format (Electronic vs. Manual) across age groups, adjusted for vaccination clinic availability; (B) predicted probability by vaccination clinic availability (Available vs. Not available) across age groups, adjusted for prescription format; (C) predicted probability by prescription format stratified by vaccine type (NIP vs. non-NIP), adjusted for vaccination clinic availability. Error bars represent 95% confidence intervals. Abbreviations: NIP, National Immunization Program; CI, confidence interval. Interaction P-values: age × prescription format, $P < 0.001$; age × vaccination clinic, $P = 0.349$; prescription format × vaccine type, $P < 0.001$.

Supplementary Figure S2. Predicted same-day conversion probability: interaction effects

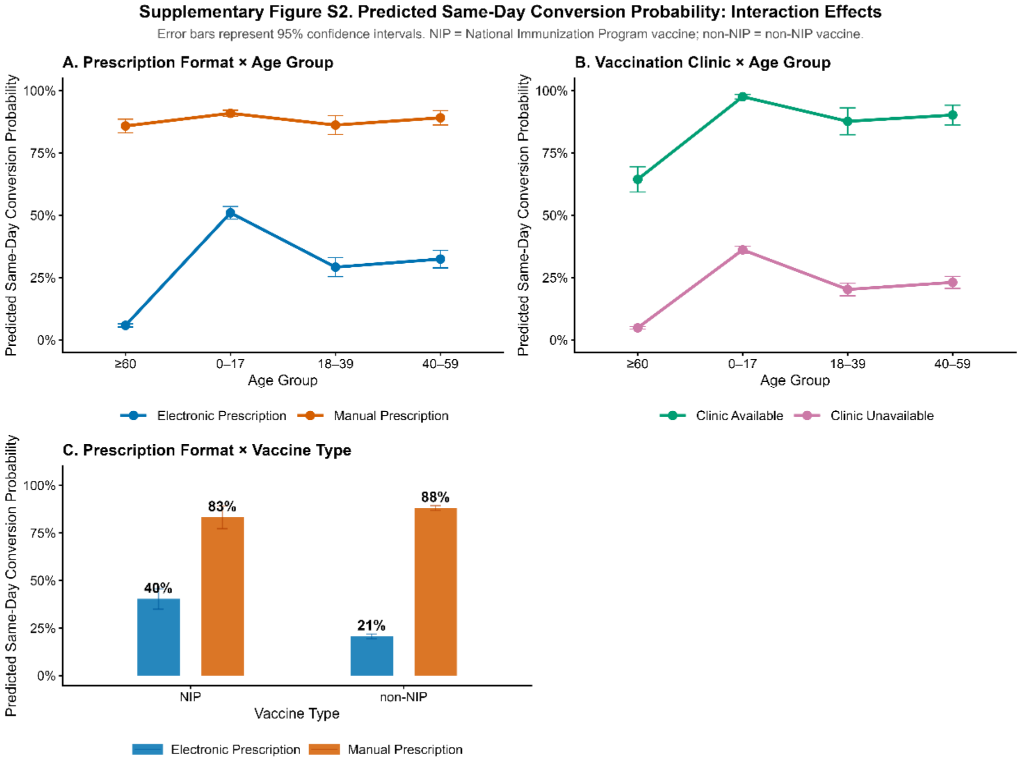

Figure S2 footnotes: Panels show predicted probabilities (with 95% CIs) derived from estimated marginal means (EM Means) based on Firth-corrected logistic regression models, with all prescriptions as the denominator. (A) predicted probability by prescription format (Electronic vs. Manual) across age groups, adjusted for vaccination clinic availability; (B) predicted probability by vaccination clinic availability (Available vs. Not available) across age groups, adjusted for prescription format; (C) predicted probability by prescription format stratified by vaccine type (NIP vs. non-NIP), adjusted for vaccination clinic availability. Error bars represent 95% confidence intervals. Abbreviations: NIP, National Immunization Program; CI, confidence interval. Interaction P-values: age × prescription format, $P < 0.001$; age × vaccination clinic, $P = 0.003$; prescription format × vaccine type, $P < 0.001$.

Supplementary Figure S3. Non-same-day conversion timeliness: interaction effects

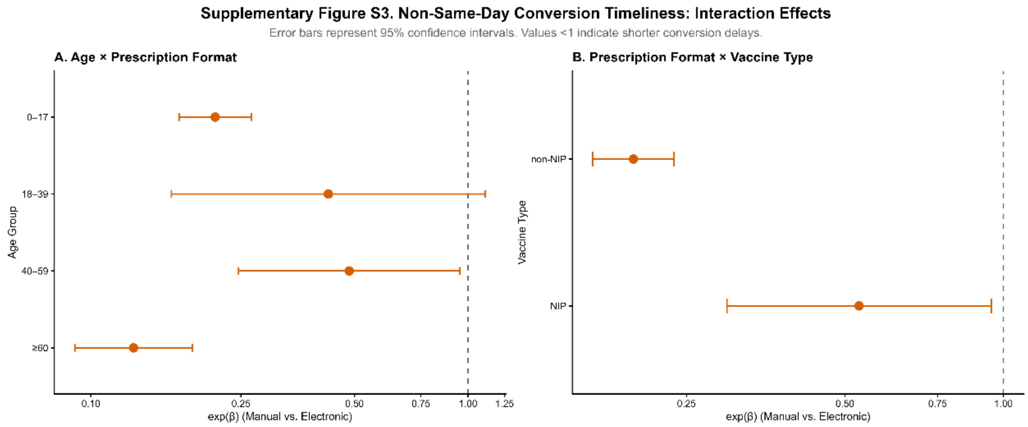

Figure S3 footnotes: Forest plots showing exp(β) and 95% CI for manual prescriptions (vs. electronic prescriptions) on log-transformed time to conversion, derived from estimated marginal means (EM Means) based on linear regression models. (A) stratified by age group; (B) stratified by vaccine type (NIP vs. non-NIP). Values <1 indicate shorter conversion delays. The dashed vertical line at 1 represents the null effect. Interaction P-values: age × prescription format, F = 124.712, df1 = 3, df2 = 642, P < 0.001; prescription format × vaccine type, F = 59.937, df1 = 1, df2 = 639, P < 0.001. Abbreviations: CI, confidence interval; NIP, National Immunization Program.
